# Complete Mitochondrial Genome of *Platygyra daedalea* and Characteristics Analysis of the Mitochondrial Genome in Merulinidae

**DOI:** 10.3390/genes16030304

**Published:** 2025-03-02

**Authors:** Shuwen Jia, Tongtong Shen, Wenqi Cai, Jian Zhang, Shiquan Chen

**Affiliations:** 1 Qukou Scientific Research Base, Institute of Marine Ecology, Hainan Academy of Ocean and Fisheries Sciences, Haikou 571136, China; jiashuwen100@163.com (S.J.); 18264019882@163.com (T.S.); wenqi_cai824@163.com (W.C.); zhangjian92213@163.com (J.Z.); 2 Key Laboratory for Coastal Marine Eco-Environment Process and Carbon Sink of Hainan Province, Yazhou Bay Innovation Institute, College of Ecology and Environment, Hainan Tropical Ocean University, Sanya 572000, China; 3 Dongzhaigang, Conservation and Restoration of Seagrass Bed Resources, Hainan Observation and Research Station, Haikou 571136, China; 4 Key Laboratory of Utilization and Conservation for Tropical Marine Bioresources (Hainan Tropical Ocean University), Ministry of Education, Sanya 572022, China

**Keywords:** coral reef, divergence time, phylogenetic, gene composition

## Abstract

Background: The Merulinidae family belonging to the order Scleractinia is mainly distributed in the Indo-Pacific and Caribbean regions and often constitute the most dominant species of coral reefs. Mitochondrial genome is a key tool for studying the phylogeny and adaptation. Only a few studies have conducted the characteristics analyses of mitochondrial genome in the Merulinidae family. Methods: Therefore, we used high-throughput sequencing technology to describe the mitochondrial genome of *Platygyra daedalea*, a member of this family. Bioinformatics was used to analyze the composition characteristics of the mitochondrial genome of 10 Merulinidae species. Results: The mitochondrial genome of *P. daedalea* had a total length of 16,462 bp and a GC content of 33.0%. Thirteen unique protein-coding genes (PCGs), two transfer RNA (tRNA) genes, and two ribosomal RNA (rRNA) genes were annotated. Each species of Merulinidae had 13 unique PCGs in the mitochondrial genome. In contrast, the number of tRNAs and rRNAs significantly varied in Merulinidae species. Collinearity and gene rearrangement analyses indicated that the mitochondrial evolution of species in the Merulinidae family was relatively conserved. Divergence time analysis indicated that Merulinidae originated in the Oligocene, whereas the *Platygyra* genus originated in the Miocene. The formation and intraspecific divergence of coral species were consistent with geological changes in the ocean. Conclusions: The results of this study help better understand the characteristics of the mitochondrial genome in the Merulinidae family and provide insights into the utility of mitochondrial genes as molecular markers of phylogeny.

## 1. Introduction

Coral reefs are among the most important ecosystems on Earth owing to their high productivity and rich biodiversity. Coral reefs significantly contribute to marine fishery productivity by providing a breeding environment, habitat, and shelter for many marine organisms, while also protecting coastlines, supporting human economic development [1,2,3,4,5,6]. However, global coral reefs have undergone large-scale degradation due to human activities, climate warming, ocean acidification, and pollution [7,8,9,10]. The phylogeny and biodiversity of scleractinian corals provide critical insights for formulating effective coral reef ecosystem conservation policies [11,12].

The mitochondrial genome is typically characterized by a compact structure, strong maternal inheritance, low rates of genetic recombination, and a highly conserved gene arrangement. These features make it has been widely used for the analysis of phylogeny and biodiversity of scleractinian corals [10,13,14,15,16,17]. The family Merulinidae (Edwards and Haime, 1857) belongs to the order Scleractinia that has the highest number of genera (24) and second highest number of coral species (154) [18]. These species, mainly distributed in the Indo-Pacific and Caribbean regions, are often among the most abundant and dominant members of coral reefs [18]. Nuclear or mitochondrial markers have been used to analyze the phylogenetic relationships of a small number of species in the Merulinidae family [14,19,20]. However, limited research has been conducted on the evolutionary history of the Merulinidae.

*Platygyra daedalea* (Ellisand Solander, 1786), a species of the Merulinidae family, is widely distributed in the Indo-Pacific [21] and is the dominant species in some parts of the South China Sea [22,23]. It is a hermaphroditic and self-incompatible species that reproducess exually through broadcast spawning [24,25] and has a strong resistance to high temperature stress [26]. Mitochondrial genes have a significant impact on the metabolic pathways and functions of organisms, therefore, environmental stress may affect the evolution of mitochondrial genes and drive adaptation in species with stress resistance [16,27]. However, information about mitochondrial genome of *P. daedalea* or Merulinidae family is limited. In this study, we sequenced and assembled the mitochondrial genome of *P. daedalea* using next-generation sequencing technology, and analyzed and annotated its basic structure. Furthermore, we analyzed phylogenetic relationships and divergence time of *P. daedalea* and other species of the Merulinidae family. This study establishes a basis and reference for the exploration of mitochondrial genomes in other species of the genus *Platygyra* and provides new molecular evidence for evolutionary studies of the Merulinidae family.

## 2. Methods

In April 2022, a 5 cm^3^ *P. daedalea* sample was collected at a depth of 2m Changjiang (19.41952253° N, 108.79193102° E), Hainan Island, China. The sample was identified as *P. daedalea* based on the morphological characteristics described in China Animal Scientific Database (http://www.zoology.csdb.cn/; 28 April 2022) and Corals of the World (http://www.coralsoftheworld.org; 28 April 2022).

Sample DNA was extracted using sodium dodecyl sulfate extraction and purified on a spin column. The extracted DNA was sent to Wuhan Beina Biotechnology Co., Ltd. for second-generation and third-generation sequencing. For second-generation sequencing, the library was prepared according to TruSeq DNA Sample Preparation Guide (Illumina, 15026486 Rev.C; San Diego, CA, USA). After the library was qualified, Illumina NovaSeq 6000 platform was used for double-ended sequencing with a read length of 150 bp. For third-generation sequencing, the library was prepared using Ligation Sequencing Kit (Oxford Nanopore Technologies, Oxford, UK). After the library was qualified, PromethION sequencer (Oxford Nanopore Technologies, Oxford, UK) was used for sequencing.

Raw data were filtered using fastp v 0.20.0 (https://github.com/OpenGene/fastp; 4 November 2023) [28].The mitochondrial genome of *P. daedalea* was assembled using a mixed assembly strategy. First, MitoZ v 3.6 [29] was used to assemble non-reference genome coral mitochondria from second-generation DNA sequencing data, generating the assembly of long scaffolds. Subsequently, GetOrganelle v 1.64 [30] was used to assemble reference mitochondria from second-generation DNA sequencing data, resulting in the assembly of long scaffolds, the MitoZ v 3.6 assembly results were used as a reference. Then, the second-generation sequencing assembly results were used as reference sequences to filter the third-generation raw sequencing data using Minimap2 v 2.28 [31], and the third-generation DNA sequencing data were assembled into coral mitochondria using Flye v 2.9.2-b1786 [32] to obtain the third-generation sequencing assembly results. The assembly results of the second- and third-generation sequencing were aligned, the starting point alignment was readjusted, and the assembly results of the second-generation sequencing were used as the basis to extend the third-generation sequencing data to obtain a circular coral mitochondrial genome. The mitochondrial genome of *P. daedalea* was visualized using OGDRAW v 1.3.1 [33] and annotated using MitoZ v 3.6 and the MFannot online tool (https://megasun.bch.umontreal.ca/apps/mfannot/; 20 November 2023) [34]. The PCGs of the mitochondrial genome were selected as a reference for *Platygyra carnosa* (Veron, 2000), a closely related species, and the annotation was manually checked for accuracy. Protein-coding sequences of the genomes were extracted using PhyloSuite v 1.2.2 [35]. Codon preference analysis was performed on the PCGs of the mitochondrial genome using Codonw v1.3 (https://codonw.sourceforge.net/; 4 December 2023) [36], and relative synonymous codon usagevalues were calculated. The results were visualized using the relative synonymous codon usage, drawing on the online bioinformatic cloud platform of Nanjing Jisi Huiyuan Biotechnology Co., Ltd., Nanjing, China.

MISA (v. 2.1) (https://webblast.ipk-gatersleben.de/misa/; 11 December 2023) [37], TRF (v. 4.09) (https://tandem.bu.edu/trf/trf.unix.help.html; 11 December 2023) [38], and REPuter network servers (https://bibiserv.cebitec.uni-bielefeld.de/reputer/; 11 December 2023) [39] were used separately to identify repetitive sequences including microsatellite sequence, tandem, and scattered duplications. The results were visualized using Microsoft Excel (2021) and Circos v. 0.69-9 [40].

Mitochondrial sequences of 47 scleractinian species were downloaded from the GenBank database, with *Corallimorphus profundus* (Moseley, 1877) and *Corynaxis californica* (Carlgren, 1936) as outgroups [41]. Thus, overall, in combination with mitochondrial DNA (mtDNA) sequences of *P. daedalea* that were obtained in this study, 50 mitochondrial sequences were analyzed. MUSCLE v3.8.1551 [42] was used to compare the 13 PCGs (*ATP6*, *ATP8*, *COX1*, *COX2*, *COX3*, *CYTB*, *ND1*, *ND2*, *ND3*, *ND4*, *ND4L*, *ND5*, and *ND6*) in the 50 samples, and Gblocks 0.91b [43] was used to remove the ambiguous regions. The processed PCGs were linked together to construct the phylogenetic tree. IQ-TREE v. 1.6.12 [44] was used for the maximum likelihood (ML) tree inference. The detection of base replacement models used IQ-TREE ModelFinder algorithm to select the optimal model. According to the Bayesian information criterion (BIC), TVM+F+I+G4 was the optimal substitution model. Bootstrap was set to Ultrafast, Num of bootstrap was set to 100,000, and the sampling frequency was 1000. The Bayesian tree (BI) used four Markov Chain Monte Carlo algorithms to run 100,000 generations simultaneously with a sampling frequency of 1000 and subsequent removal of 25% of the aging samples. The BI was implemented using PhyloSuite v1.2.2 [35].

Species divergence time was inferred using the sequences processed with MUSCLE v3.8.1551 [42] and Gblocks 0.91b [43], as above. Three sets of fossil nodes were extracted from Timetree (http://www.timetree.org/; 15 December 2023) as reference nodes for divergence time analysis. The differentiation time of *Acropora horrida* (Dana, 1846) and *Montipora cactus* (Bernard, 1897) fossils was 26.0–72.0 Ma, and the median time was 50 Ma. The fossil differentiation time of *A. horrida* and *Orbitella faveolate* (Ellis and Solander, 1786) was 168.8–415 Ma, and the median time was 264 Ma. The fossil differentiation time of *A. horrida* and *C. profundus* was 263.1–451.7 Ma, with a median time of 363 Ma. Divergence time inference was performed using the above mentioned 13 PCG sequences from 50 species using BEAST v2.6.2 [45]. Strict Clock was used as the inference method, and 10,000,000 generation inspections were performed during the Markov Chain Monte Carlooperation. During the process of the collegiate tree, statistical analyses were conducted for every 1000 generations. The top 25% of the trees were discarded as aging trees, and the remaining trees were considered to indicate the branching time represented by the tree structure and nodes. Tracer v 1.7.1 [46] was used to view the tree file with an effective sample size exceeding 200. Finally, FigTree v1.4.4 was used to view the tree results and adjust them.

To further analyze the mitochondrial genome of Merulinidae, we performed a collinearity analysis and selective pressure analysis using the 10 species for which the mitochondrial genomes have been published. A perl script was used to obtain the gene arrangement order of the 11 species (10 Merulinidae species and *Echinophyllia aspera* (Ellis and Solander, 1786) as the outgroup), and Adobe Illustrator CS6 v 1.0 was used to draw a gene rearrangement map. Genome sequence ML trees of the 11 species were constructed using IQ-TREE v. 1.6.12 [44]. The optimal model was selected by detection of base replacement models using the IQ-TREE ModelFinder algorithm. According to the BIC, the optimal substitution model was TVM+F+R2. Bootstrap was set to Ultrafast, Num of bootstrap was set to 100,000, and the sampling frequency was 1000. The collinearity analysis graph was obtained using AliTV v 1.0.6 (https://alitvteam.github.io/AliTV/d3/AliTV.html; 14 February 2024) [47].

Using the mitochondrial genome of *P. daedalea* as a reference sequence, mitochondrial genome sequences of all 11 selected species were compared using Brig v1.6. The 13 PCGs were aligned and formatted using ParaAT v 2.0 [48] with default parameters. Subsequently, the Ka/Ks Calculator v2.0 [49] was used to calculate the Ka, Ks, and Ka/Ks values based on the Yang–Nielsen method. In addition, the mitochondrial genomes were compared using MAFFT v 7.429 [50], and the nucleotide variation (Pi) of the mitochondrial genome was analyzed using DnaSp v6 [51] for sliding window analysis using the *P. daedalea* genome as a reference. The window length and step size were set to 300 bp and 25 bp, respectively.

## 3. Results

### 3.1. Analysis of Mitochondrial Genome Characteristics

**Figure 1:**
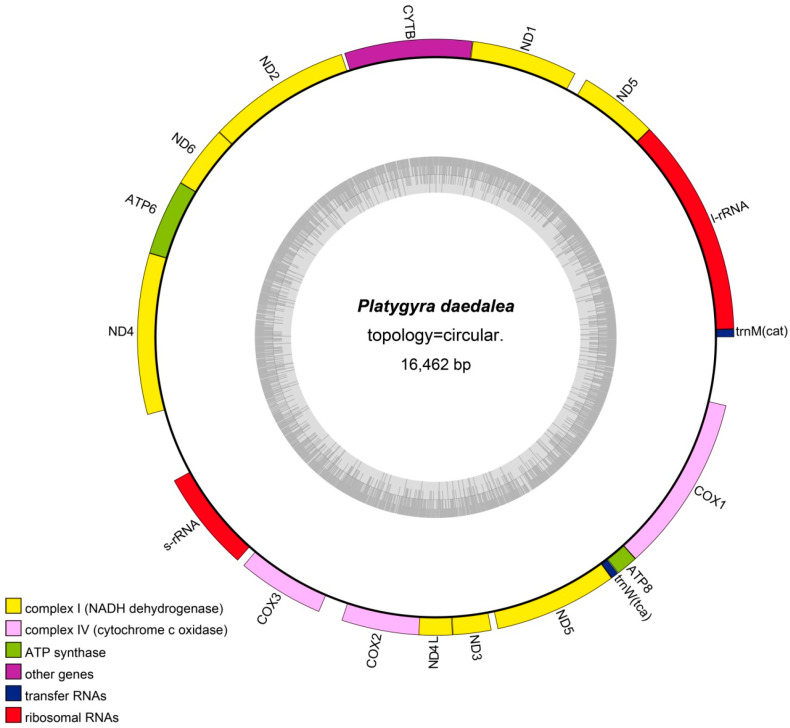
Circular map of the complete mitochondrial genome of *Platygyra daedalea*. The outer colored circle represents the mitochondrial genome, with different colors indicating distinct gene categories. The inner circle displays GC content (dark gray) and AT content (light gray).

**Figure 2:**
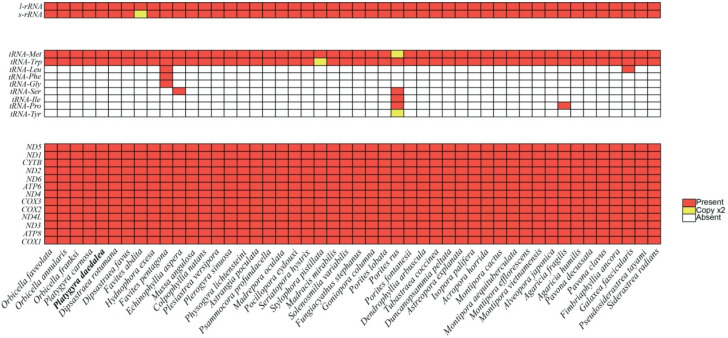
Comparison of the mitochondrial genes of the 48 scleractinian coral species. White rectangles indicate gene absence, red rectangles indicate one gene copy, and yellow rectangles indicate two gene copies.

The total length of the mitochondrial genome of *P. daedalea* was 16,462 bp, with a GC content of 33.0%. Thirteen unique PCGs, two tRNA genes, and two rRNA genes were annotated (Figure 1). The PCGs included two ATP synthase genes (*ATP6* and *ATP8*), seven NADH dehydrogenase genes (*ND1*, *ND2*, *ND3*, *ND4*, *ND4l*, *ND5*, and *ND6*), three cytochrome C oxidase genes (*COX1*, *COX2*, and *COX3*), and one panthenol-cytochrome C reductase gene (*CYTB*). Comparisons of mitochondrial genes of *P. daedalea* with those of 47 other scleractinian species revealed that the mitochondrial structure of scleractinian corals was similar (Figure 2). Forty-eight scleractinian coral species had 13 unique PCGs, and 47 scleractinian coral species had two rRNAs. However, the number of tRNAs greatly varied among coral species: *Favites pentagona* (Esper, 1790), *E. aspera*, *Porites rus* (Forskål, 1775), *Agaricia fragilis* (Dana, 1846), *Galaxea fascicularis* (Linnaeus, 1767), and *Stylophora pistillata* (Esper, 1792)contained more than two tRNAs.

**Figure 3:**
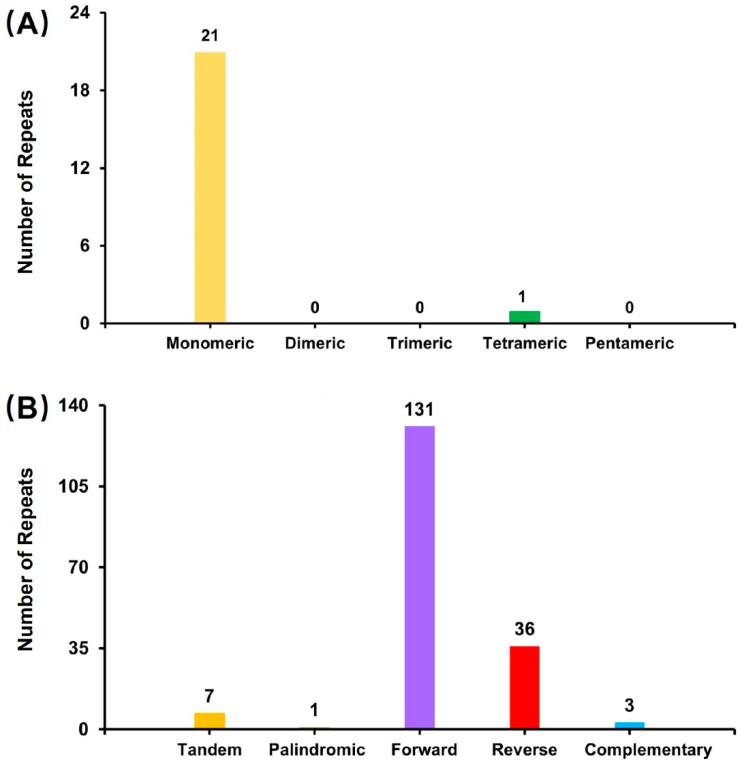
The type of microsatellite (**A**) and interspersed repeated sequences (**B**).

Repeated sequence analysis of the *P. daedalea* mitochondrial genome revealed 22 simple sequence repeats (SSRs). Among these, 21 SSRs had single-base repeats, accounting for 95.45% of all SSRs (Figure 3A). A total of 171 pairs of interspersed repeated sequences (IRSs), each with a length of 25 nucleotides or more, were observed in the mitochondrial genome of *P. daedalea* (Figure 3B).

### 3.2. Phylogenetic Analysis of Mitochondrial Genome

**Figure 4:**
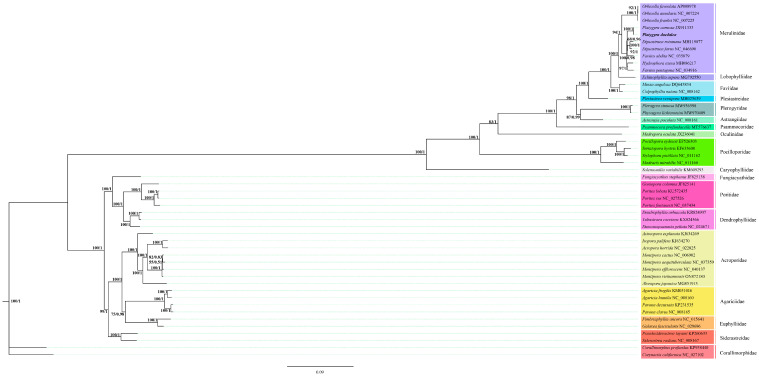
Phylogenetic tree of 48 species of Scleractinia based on maximum likelihood and Bayesian inference analyses of 13 unique protein-coding genes. Numbers at the significant nodes represent maximum likelihood bootstrap values and Bayesian posterior probabilities; the scale label presents the distance scale, with different background colors representing different families; and the numbers in parentheses correspond to GenBank accession numbers of related strains.

We applied BI and ML methods to construct a phylogenetic tree by using nucleotide sequences of 13 PCGs in the mitochondrial genome of 48 scleractinian species from 17 families (Figure 4). The topological structures of the phylogenetic trees constructed using the BI and ML methods were similar. Forty-eight spieces were divided into two major branches, one belonging to the robust and the other belonging to the complex branch. The 10 Merulinidae species were divided into two branches, one consisting of *Orbicella* and the other consisting of *Platygyra*, *Dipsastraea*, *Favites*, and *Hydrophora*.

**Figure 5:**
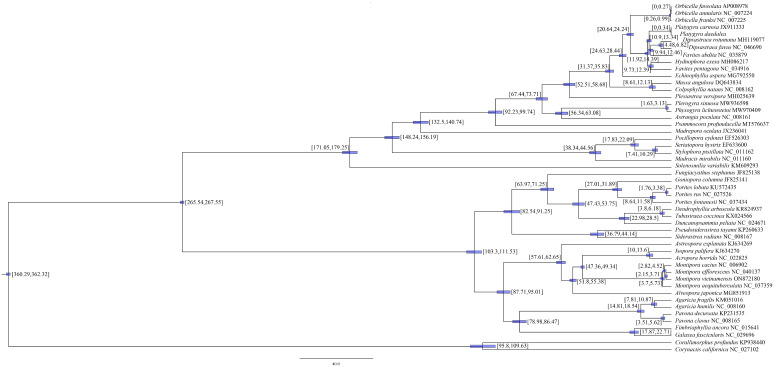
Divergence times of 48 species of Scleractinia inferred using BEAST. Numbers above the branches indicate divergence time. The size of the blue band represents the 95% higher posterior density of divergence time.

Based on the mtDNA sequences of 13 PCGs, we estimated the divergence time and concluded that the family Merulinidae originated in the Oligocene (24.63–28.44 Ma), and the genus *Platygyra* originated in the Miocene (10.9–13.34 Ma; Figure 5).

**Figure 6:**
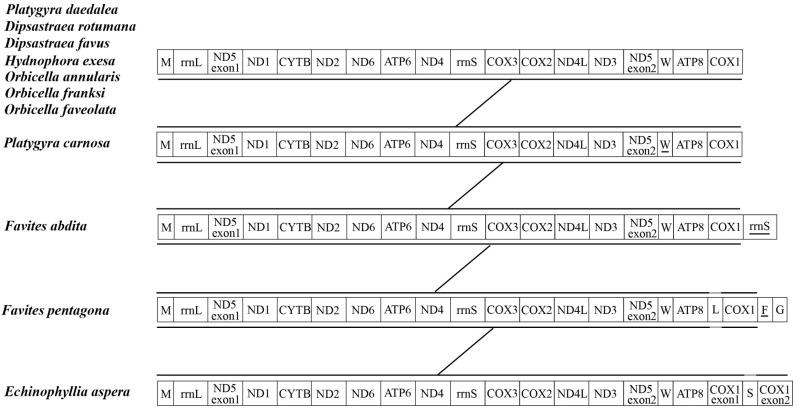
Gene arrangement analysis of the mitochondrial genome of the 10 species of Merulinidae. During the evolutionary process, the order of arrangement of mitochondrial genes showed varying degrees of collinearity in different species, and some adjacent genes tended to form highly conserved gene clusters. The variation in mtDNA between different genera and species in the Merulinidae family was relatively low and the arrangement and direction of conserved sequence segments were relatively consistent with a high degree of collinearity; this indicated that they underwent less recombination during the independent evolutionary process of each genus and species (Figure 7).

Gene rearrangement analysis showed that the genes of Merulinidae corals were arranged in a relatively consistent order, with the exception of those of *P. carnosa*, *F.abdita*, and *F. pentagona* (Figure 6). The mitochondrial genes of Merulinidae species were relatively conserved.

Mitochondrial genome sequences have been successfully used to clarify the evolutionary relationships between various biological populations. In this study, there was a significant difference between the topological structure inferred from the collinearity analysis and that inferred from the phylogenetic analysis. In the analysis of collinearity, the outgroup *E. aspera* entered the family Merulinidae. The two species within the genus *Dipsastraea* did not cluster together or with *F. abdita*. However, species within the *Platygyra* and *Orbicella* genera were clustered together, as were *F. pentagona* and *Hydnophora exesa*, these results were consistent with the phylogenetic tree results based on the analysis of PCGs.

**Figure 8:**
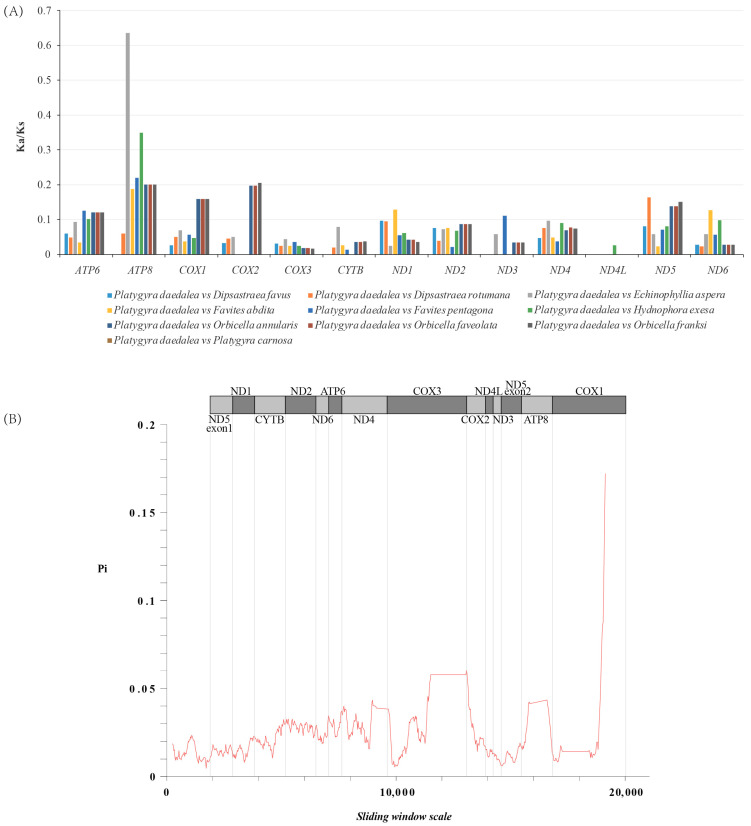
(**A**) Ka/Ks ratios of protein-coding genes in 13 unique protein-coding genes of *Platygyra daedalea.* (**B**) Sliding window analysis of the mitochondrial genome of the 10 species of Merulinidae using the DnaSP v6 program.

The Ka/Ks values were all under 1, indicating that mitochondrial genes of *P. daedalea* and other corals in the family Merulinidae were highly conserved during evolution. *COX1* showed the highest nucleotide diversity, followed by *COX3*, whereas ND5 showed the highest conservation (Figure 8; Table S1).

## 4. Discussion

### 4.1. Characteristics of the Merulinidae mtDNA Structure

In this study, we compared the PCG, tRNAs, and rRNAs of 48 scleractinian corals and found that the number and gene type of PCGs and rRNAs of scleractinian corals were relatively conserved, but the number and type of tRNAs greatly varied. The difference between the mtDNA of scleractinian coral and that of other metazoan species is mainly in the tRNAs. For example, most arthropods and vertebrates have 37 coding genes, 22 of which are tRNAs [52,53]. Most scleractinian corals have only two tRNAs, namely, tRNA-Met and tRNA-Trp [14,41,54]. Only a few scleractinian corals have multiple tRNAs. In previous studies, rRNAs were also found to be relatively conserved, while the number and structure of tRNAs considerably varied [15]. The mtRNAs of the Merulinidae species also conform to this structural feature, and there are only two tRNAs in eight of the 10 Merulinidae species analyzed in this study. Corals are one of the most basic metazoans, and other metazoans may have evolved to have more tRNAs to perform more complex mitochondria functions.

The number of PCGs in most Arthropods and vertebrates is 13, but there are changes in the number of PCGs in many species of cnidarians. Moreover, the cnidarian mtDNA encodes many additional PCGs, such as the octopus specific mutS protein [15]. In this study, the number of PCGs of scleractinian coral, as a member of the cnidarians species, is relatively conservative, and the number of PCGs of the 48 scleractinian coral species is 13, which is similar to that in other higher metazoans. The mitochondrion genomes of Merulinidae species are basically consistent in terms of genome structure, gene number, type, and order. There is a significant rearrangement of genes in the evolution of the spinocytic animals’ mitochondrion genomes, contrasting with the high level of conservation in the Merulinidae species. This conservation indicates evolutionary constraints that are prevalent in the mitochondrion genomes of scleractinian corals [14].

### 4.2. Intraspecific Divergenceof Merulinidae

The process and mechanism of speciation and divergence is the key to understanding the phenomena of biodiversity in nature, and the time of species divergence is the key to understanding the mechanism of speciation. Park et al. [15] studied the molecular divergence time of Cnidaria based on the mitochondrial DNA sequences of 13 PCGs, and the results showed that cnidarians originated 741 Ma. However, although divergence time analysis and fossil evidence provide insights into the phylogenetic relationships within some taxa of scleractinian corals, the most recent common ancestors of coral families and genera remain unknown [14,55,56]. In this study, our results suggest that Merulinidae originated in the Oligocene, *Platygyra* originated in the Miocene, and intraspecific differentiation of *Platygyra* occurred in the Pleistocene. We estimate that the time scale of the genetic differences is consistent with the geological and climatic conditions of the respective geographical regions. For example, the South China Sea experienced rupture in the eastern part during the Oligocene and Miocene (32 Ma) and ridge transition (23.5 Ma), change in expansion direction (20 Ma), and cessation of expansion (15.5 Ma), which were consistent with the origin time of Merulinidae and *Platygyra* [57]. During the late Oligocene to the early Miocene, scleractinian corals exhibited rich diversity, coinciding with a globally stable cold period in climate history [58,59]. The diversity of corals decreased in the Late Miocene, during which many major geological and climatic changes took place around the globe, and many terrestrial animals and plants also underwent speciation and divergence. Further, Pleistocene climate change is an important factor in the formation and divergence of numerous terrestrial and marine fauna [58,59,60,61,62]. Therefore, the speciation and divergence of scleractinian corals are closely related to paleoclimatic and paleogeological changes.

## 5. Conclusions

Among the published studies of scleractinian corals, few studies reported the evolutionary analyses at the family, genus, and species levels, and most corals lack universal molecular markers with suitable resolution. The entire mitochondrial genome is widely used as a molecular marker in the systematics of animal and plants pecies. In the present study, we used a combination of second- and third-generation sequencing approaches to sequence the complete mtDNA of *P. daedalea*, which is the second complete genome of the genus *Platygyra*. By comparing the mitochondria of 48 scleractinian corals, we showed that their gene structure was relatively conserved. Thirteen unique PCGs and two rRNAs were found in the genome of scleractinian corals, however, the number of tRNAs greatly varied among different species. The molecular clock model estimated that Merulinidae originated in the Oligocene, *Platygyra* originated in the Miocene, and intraspecific differentiation of *Platygyra* occurred in the Pleistocene. In mtDNA genomes of 10 Merulinidae family species, the Ka/Ks values were less than 1, the variation in mtDNA between genera and species was relatively low, the order and direction of conserved sequence segments were relatively consistent, and the degree of collinearity was high, indicating that mtDNA underwent less extensive reorganization during the independent evolution of each genus and species. The present study provides new insights for the systematics and evolutionary studies of the mitochondrial genomes of *P. daedalea* and members of the Merulinidae family.

## Figures and Tables

**Figure 7:**
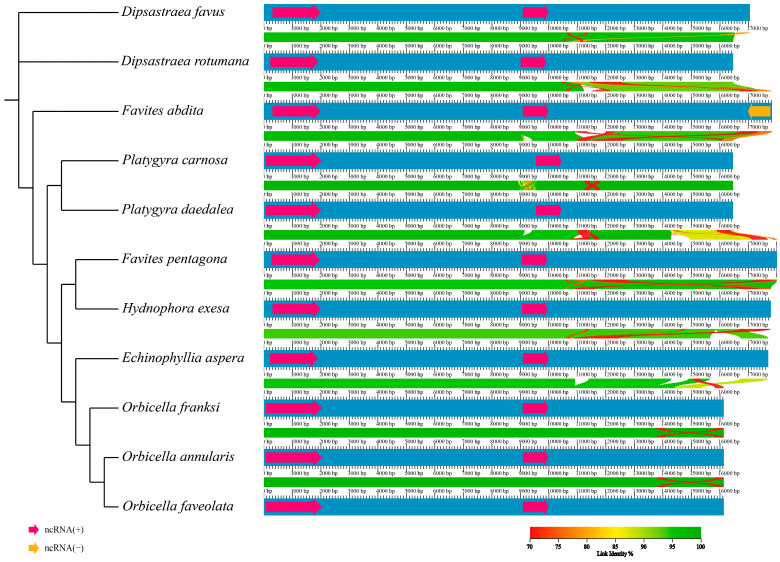
Collinearity analysis of the mitochondrial genome of the 10 species of Merulinidae.

## Data Availability

The mitochondrial genome sequence and SRA data of *Platygyra daedalea* reported in this study were deposited in GenBank under accession number PP856480, SAMN45919465 and SAMN45919466.

## Supplementary Materials

The following supporting information can be downloaded at: https://www.mdpi.com/article/10.3390/genes16030304/s1, Table S1: ka/ks value list of *Platygyra daedalea*.

## References

[1] Guohua C., Liangmin H., Hankui W., Hui H., Yehui T., Si Z., Junde D. (2004). Status and prospectives of research on coral reef ecosystem primary production. Acta Ecol. Sin..

[2] Hoegh-Guldberg O. (1999). Climate change coral bleaching and the future of the world’s coral reefs. Mar. Freshwater Res..

[3] Moberg F., Folke C. (1999). Ecological goods and services of coral reef ecosystems. Ecol. Econ..

[4] Porter J.W., Tougas J.I., Levin S.A. (2001). Encyclopedia of Biodiversity.

[5] Wilkinson C.C.R. (2004). Status of Coral Reefs of the World, Vol. 2004.

[6] Yang Y.W., Chen A.C. (2002). Evolutionary ecology of zooxanthellae diversity and coral bleaching. J. Biol. Sci..

[7] Bellwood D.R., Pratchett M.S., Morrison T.H., Gurney G.G., Hughes T.P., Álvarez-Romero J.G., Day J.C., Grantham R., Grech A., Hoey A.S. (2019). Coral reef conservation in the Anthropocene: Confronting spatial mismatches and prioritizingfunctions. Biol. Conserv..

[8] Hughes T.P. (1994). Catastrophes, phase shifts, and large-scale degradation of a Caribbean coral reef. Science.

[9] Pratchett M.S., Hoey A.S., Wilson S.K. (2014). Reef degradation and the loss of critical ecosystem goods and services provided by coral reef fishes. Curr. Opin. Environ. Sustain..

[10] Rivas N., Gómez C.E., Millán S., Mejía-Quintero K., Chasqui L. (2023). Coral reef degradation at an atoll of the Western Colombian Caribbean. PeerJ.

[11] Huang D., Roy K. (2015). The future of evolutionary diversity in reef corals. Philos. Trans. R. Soc. B Biol. Sci..

[12] Hughes T.P., Barnes M.L., Bellwood D.R., Cinner J.E., Cumming G.S., Jackson J.B., Kleypas J., Van De Leemput I.A., Lough J.M., Morrison T.H. (2017). Coral reefs in the Anthropocene. Nature.

[13] Feng H., Lv S., Li R., Shi J., Wang J., Cao P. (2023). Mitochondrial genome comparison reveals the evolution of cnidarians. Ecol. Evol..

[14] Niu W.T., Xiao J.G., Tian P., Yu S.E., Guo F., Wang J.J., Huang D. (2020). Characterization of the complete mitochondrial genome sequences of three Merulinidae corals and novel insights into the phylogenetics. Peer J.

[15] Park E., Hwang D.S., Lee J.S., Song J.I., Seo T.K., Won Y.J. (2012). Estimation of divergence times in cnidarian evolution based onmitochondrial protein-coding genes and the fossil record. Mol. Phylogenet. Evol..

[16] Ramos N.I., Deleo D.M., Horowitz J., McFadden C.S., Quattrini A.M. (2023). Selection in coral mitogenomes, with insights into adaptations in the deep sea. Sci. Rep..

[17] Seiblitz I.G., Vaga C.F., Capel KC C., Cairns S.D., Stolarski J., Quattrini A.M., Kitahara M.V. (2022). Caryophylliids (Anthozoa, Scleractinia) and mitochondrial gene order: Insights from mitochondrial and nuclear phylogenomics. Mol. Phylogenet. Evol..

[18] Dai C.F., Cheng Y.R. (2020). Corals of Taiwan, Vol. 1: Scleractinia Fauna.

[19] Fukami H., Budd A.F., Paulay G., Solé-Cava A., Allen Chen C., Iwao K., Knowlton N. (2004). Conventional taxonomy obscures deep divergence between Pacific and Atlantic corals. Nature.

[20] Huang D.W., Licuanan W.Y., Baird A.H., Fukami H. (2011). Cleaning up the ‘Bigmessidae’: Molecular phylogeny of scleractinian corals from Faviidae, Merulinidae, Pectiniidae and Trachyphylliidae. BMC Evol. Biol..

[21] Veron J.E.N. (2000). Corals of the World.

[22] Gu Q., Li H., Qian J., Liu J., Shi Y., He K., Li Y., Yin A. (2017). Species composition and distribution of Scleractinia Coral in the port of Hainan Dazhou Island. Nat. Sci. J. Hainan Univ..

[23] He X.N., Wu Z.J., Chen M., Chen S.Q., Zhang M.J., Li Y.C. (2018). Distribution and healthy evaluation of corals surrounding Qizhou Islands in Hainan. Wetland Sci. Manag..

[24] Babcock R.C., Bull G.D., Harrison P.L., Heyward A.J., Oliver J.K., Wallace C.C., Willis B.L. (1986). Synchronous spawnings of 105 scleractinian corals pecieson the Great Barrier Reef. Mar. Biol..

[25] Miller K.J., Ayre D.J. (2008). Population structure is not a simple function of reproductive mode and larval type: Insights from tropical corals. J. Anim. Ecol..

[26] McClanahan T.R., Baird A.H., Marshall P.A., Toscano M.A. (2004). Comparing bleaching and mortality responses of hard corals between southern Kenya and the Great Barrier Reef, Australia. Mar. Pollut. Bull..

[27] Wei Z., Ta K., Zhang N., Liu S., Meng L., Liu K., Cai C., Peng X., Shao C. (2024). Molecular phylogenetic relationships based on mitochondrial genomes of novel deep-seacorals (Octocorallia: Alcyonacea): Insights into slow evolution and adaptation to extreme deep-sea environments. Zool. Res..

[28] Chen S.F., Zhou Y.Q., Chen Y.R., Gu J. (2018). fastp: An ultra-fast all-in-one FASTQ preprocessor. Bioinformatics.

[29] Meng G.L., Li Y.Y., Yang C.T., Liu S.L. (2019). MitoZ: A tool kit for animal mitochondrial genome assembly, annotation and visualization. Nucleic Acids Res..

[30] Jin J.-J., Yu W.-B., Yang J.-B., Song Y., de Pamphilis C.W., Yi T.-S. (2020). Get Organelle: A fast and versatile tool kit for accurate de novo assembly of organelle genomes. Genome Biol..

[31] Li H. (2018). Minimap2: Pairwisea lignment for nucleotide sequences. Bioinformatics.

[32] Kolmogorov M., Yuan J., Lin Y., Pevzner P.A. (2019). Assembly of long, error-prone reads using repeat graphs. Nat. Biotechnol..

[33] Greiner S., Lehwark P., Bock R. (2019). Organellar Genome DRAW (OGDRAW) version 1.3.1: Expanded toolkit for the graphical visualization of organellar genomes. Nucleic Acids Res..

[34] Lang B.F., Beck N., Prince S., Sarrasin M., Rioux P., Burger G. (2023). Mitochondrial genome annotation with MFannot: A critical analysis of gene identification and gene model prediction. Front. Plant Sci..

[35] Zhang D., Gao F., Jakovlić I., Zou H., Zhang J., Li W.X., Wang G.T. (2020). PhyloSuite: An integrated and scalable desktop platform for streamlined molecular sequence data management and evolutionary phylogenetics studies. Mol. Ecol. Resour..

[36] Sharp P.M., Tuohy T.M., Mosurski K.R. (1986). Codon usage in yeast: Cluster analysis clearly differentiates highly and lowly expressed genes. Nucleic Acids Res..

[37] Beier S., Thiel T., Münch T., Scholz U., Mascher M. (2017). MISA-web: A web server for microsatellite prediction. Bioinformatics.

[38] Benson G. (1999). Tandem repeats finder: A program to analyze DNA sequences. Nucleic Acids Res..

[39] Kurtz S., Choudhuri J.V., Ohlebusch E., Schleiermacher C., Stoye J., Giegerich R. (2001). REPuter: The manifold applications of repeat analysis on a genomic scale. Nucleic Acids Res..

[40] Zhang H., Meltzer P., Davis S. (2013). RCircos: An R package for Circos 2D track plots. BMC Bioinform..

[41] Tian P., Jia Z.Y., Cao B., Wang W., Xiao J.G., Niu W.T. (2022). Complete mitochondrial genome sequences of *Physogyra lichtensteini* (Milne Edwards & Haime, 1851) and *Plerogyra sinuosa* (Dana,1846) (Scleractinia, Plerogyridae): Characterisation and phylogenetic analysis. ZooKeys.

[42] Edgar R.C. (2004). MUSCLE: Multiple sequence alignment with high accuracy and high throughput. Nucleic Acids Res..

[43] Castresana J. (2000). Selection of conserved blocks from multiple alignments for their use in phylogenetic analysis. Mol. Biol. Evol..

[44] Nguyen L.T., Schmidt H.A., VonHaeseler A., Minh B.Q. (2015). IQ-TREE: A Fast and Effective Stochastic Algorithm for Estimating Maximum-Likelihood Phylogenies. Mol. Biol. Evol..

[45] Bouckaert R., Vaughan T.G., Barido-Sottani J., Duchêne S., Fourment M., Gavryushkina A., Heled J., Jones G., Kühnert D., De Maio N. (2019). BEAST2.5: An Advanced Software Platform for Bayesian Evolutionary Analysis. PLoS Comput. Biol..

[46] Rambaut A., Drummond A.J., Xie D., Baele G., Suchard M.A. (2018). Posterior summarisation in Bayesian phylogenetics using Tracer1.7. Syst. Biol..

[47] Ankenbrand M.J., Hohlfeld S., Hackl T., Förster F. (2017). AliTV—Interactive visualization of whole genome comparisons. Peer J Comput. Sci..

[48] Zhang Z., Xiao J., Wu J., Zhang H., Liu G., Wang X., Dai L. (2012). ParaAT: A parallel tool for constructing multiple protein-coding DNA alignments. Biochem. Biophys. Res. Commun..

[49] Wang D., Zhang Y., Zhang Z., Zhu J., Yu J. (2010). KaKs_Calculator 2.0: A tool kit incorporating gamma-series methods and sliding window strategies. Genom. Proteom. Bioinf..

[50] Katoh K., Standley D.M. (2016). A simple method to control over-alignment in the MAFFT multiple sequence alignment program. Bioinformatics.

[51] Rozas J., Ferrer-Mata A., Sánchez-DelBarrio J.C., Guirao-Rico S., Librado P., Ramos-Onsins S.E., Sánchez-Gracia A. (2017). DnaSP6: DNA sequence polymorphism analysis of large datasets. Mol. Biol. Evol..

[52] Boore J.L. (1999). Animal mitochondrial genomes. Nucleic Acids Res..

[53] Lin X.X., Dong W.G. (2024). Research progress on mitochondrial genomes of Pulicinae. Chin. J. Parasitol. Parasit. Dis..

[54] Tian P., Wang W., Xu Z., Cao B., Jia Z., Sun F., Xiao J., Niu W. (2023). The Complete mitochondrial Genome of *Homophyllia bowerbanki* (Scleractinia, Lobophylliidae): The First Sequence for the Genus Homophyllia. Genes.

[55] Gold D.A., Runnegar B., Gehling J.G., Jacobs D.K. (2015). Ancestral state reconstruction of ontogeny supports a bilaterian affinity for Dickinsonia. Evol. Dev..

[56] Simpson C., Kiessling W., Mewis H., Baron-Szabo R.C., Müller J. (2011). Evolutionary diversification of reef corals: A comparison of the molecular and fossil records. Evolution.

[57] Yang T.Y., Tang Y., Ren J.Y., Chao P. (2023). The rift evolution and magmatic-tectonic-stratigraphic records of episodic seafloorspreading at South west Sub-basin of the South China Sea. Earth Sci..

[58] Schuster F., Wielandt U. (1999). Oligocene and Early Miocene coral faunas from Iran: Palaeoecology and palaeobiogeography. Int. J. Earth Sci..

[59] Vertino A., Stolarski J., Bosellini F.R., Taviani M. (2014). Mediterranean corals through time: From Miocene to Present. The Mediterranean Sea: Its History and Present Challenges.

[60] Fan Q.C., Xu Z.K., Sun T.Q., Li T.G., Chang F.M. (2022). Sediment source-to-sink processes of the southeastern Indian Ocean during the late Eo-cene-Oligocene and their potential significance for paleoclimate. Bull. Geol. Sci. Technol..

[61] Jia S.W., Zhang M.L. (2021). Introgression of phylogeography lineages of *Convolvulus gortschakovii* (Convolvulaceae) in the northwest China. Plant Syst. Evol..

[62] Jia S., Xu L., Geng X., Zhang H. (2022). Comparative evolutionary history of two closely related desert plant, *Convolvulu stragacanthoide* and *Convolvulus gortschakovii* (Convolvulaceae) from northwest China. Ecol. Evol..

